# Factors associated with IgG levels in adults with IgG subclass deficiency

**DOI:** 10.1186/s12865-021-00447-3

**Published:** 2021-08-09

**Authors:** James C. Barton, Jackson Clayborn Barton, Luigi F. Bertoli, Ronald T. Acton

**Affiliations:** 1 University of Alabama at Birmingham, Birmingham, AL USA; 2 Southern Iron Disorders Center, Birmingham, AL USA; 3 Department of Medicine, Brookwood Medical Center, Birmingham, AL USA; 4 Department of Microbiology, University of Alabama At Birmingham, Birmingham, AL USA

**Keywords:** IgG subclass deficiency, IgG1, IgG2, IgG3

## Abstract

**Background:**

Factors associated with IgG levels in adults with IgG subclass deficiency (IgGSD) are incompletely understood. We studied adults with IgGSD with subnormal IgG1 only, subnormal IgG1/IgG3, or subnormal IgG3 only without other subnormal IgG subclasses, IgA, or IgM. We compiled: age; sex; autoimmune condition(s) (AC); atopy; IgG, IgG subclasses, IgA, IgM; IgGsum (IgG1 + IgG2 + IgG3 + IgG4); and D (percentage difference between IgGsum and IgG). We compared attributes of patients with/without subnormal IgG (< 7.00 g/L; subnormal IgG1 subclass groups only) and analyzed IgGsum and IgG relationships. We performed backward stepwise regressions on IgG using independent variables IgG subclasses, age, and sex and on D using independent variables age and sex.

**Results:**

There were 39 patients with subnormal IgG1 only (89.7% women), 53 with subnormal IgG1/IgG3 (88.7% women), and 115 with subnormal IgG3 only (91.3% women). Fifteen patients (38.5%) and 32 patients (60.4%) in the respective subnormal IgG1 subclass groups had subnormal IgG. Attributes of patients with/without IgG < 7.00 g/L were similar, except that AC prevalence was lower in patients with subnormal IgG1 only and IgG < 7.00 g/L than ≥ 7.00 g/L (*p* = 0.0484). Mean/median IgG1 and IgG2 were significantly lower in patients with IgG < 7.00 g/L in both subnormal IgG1 subclass groups (*p* < 0.0001, all comparisons). Regressions on IgG in three subclass groups revealed positive associations with IgG1 and IgG2 (*p* < 0.0001 each association). Regressions on D revealed no significant association. IgG1 percentages of IgGsum were lower and IgG2 percentages were higher in patients with subnormal IgG1 subclass levels than subnormal IgG3 only (*p* < 0.0001 all comparisons).

**Conclusions:**

We conclude that both IgG1 and IgG2 are major determinants of IgG in patients with subnormal IgG1, combined subnormal IgG1/IgG3, or subnormal IgG3 and that in patients with subnormal IgG1 or combined subnormal IgG1/IgG3, median IgG2 levels are significantly lower in those with IgG < 7.00 g/L than those with IgG ≥ 7.00 g/L.

**Supplementary Information:**

The online version contains supplementary material available at 10.1186/s12865-021-00447-3.

## Background

Immunoglobulin (Ig) G (IgG) is the predominant of five classes of Ig (IgG, IgA, IgM, IgE, and IgD). Igs differ in heavy chain structure and effector function [1]. IgG1, the largest IgG subclass, represents ~ 60% of IgG [1, 2] and has a half-life of 21 days [3]. Antibody responses to soluble protein and membrane antigens primarily induce IgG1 [1, 4], although polysaccharides and allergens also elicit IgG1 responses [1]. In two studies, adults with frequent or severe respiratory tract infection had subnormal IgG1 (< 2 standard deviations (SD) below respective means) in the absence of subnormal levels of other IgG subclasses, subnormal IgA, or subnormal IgM [5, 6].

In normal adults, IgG2 represents ~ 30% of serum IgG [1, 2]. IgG2 activates complement less readily than IgG1 and IgG3 [1], has low affinity for Fc receptors on phagocytes (FcγR) [1], crosses the placenta less freely than other IgG subclasses [7, 8], and has a half-life of 21 days [3]. IgG2 is the predominant antibody that responds to bacterial polysaccharide antigens [9–12]. Some persons with frequent or severe respiratory tract infection have subnormal IgG2 (< 2 SD below respective means) [13–16].

In normal adults, IgG3 represents ~ 4% of serum IgG [1, 17]. IgG3 activates complement more readily than other IgG subclasses [1], has high affinity for Fc receptors on phagocytes (FcγR) [1], crosses the placenta less readily than IgG1 and IgG4 but more so than IgG2 [18], and has an overall half-life of ~ 7 days [1, 3]. Some adults with frequent or severe respiratory tract infection have subnormal IgG3 (< 2 SD below respective means) [19–21].

IgG subclass deficiency (IgGSD) is characterized by frequent or severe upper or lower respiratory tract infection, one or more subnormal IgG subclass level(s) (≤ 2 SD below respective means) unexplained by other causes, and decreased IgG response to pneumococcal polysaccharide vaccination (PPSV) [20–25]. The predominance of women with IgGSD [21, 24, 25] becomes evident at ages ≥ 16 y [22]. IgGSD occurs in ~ 1 in 10,000 persons [26]. In two studies, IgG levels were subnormal in 38% and 46% of adults with IgGSD who had subnormal IgG1 only [5, 6] and in three adults with IgGSD and subnormal IgG2 in whom IgG1 levels were not subnormal [16]. These observations suggest that both IgG1 and IgG2 are determinants of IgG in adults with IgGSD.

We sought to identify factors associated with IgG levels in adults at diagnosis of IgGSD. We performed a retrospective chart and data review to identify adults diagnosed with IgGSD in a hematology clinic who had subnormal IgG1 only, combined subnormal IgG1/IgG3, or subnormal IgG3 only at diagnosis without other subnormal IgG subclasses, subnormal IgA, or subnormal IgM. We compiled these data: age at diagnosis; sex; autoimmune condition(s) (AC); atopy; serum levels of IgG, IgG subclasses, IgA, IgM; IgGsum (IgG1 + IgG2 + IgG3 + IgG4); and the D-parameter (D), the percentage difference between IgGsum and IgG [27]. In subnormal IgG1 only or combined subnormal IgG1/IgG3 subclass groups, we compared clinical and laboratory attributes of patients with and without IgG < 7.00 g/L. In each of the three subnormal Ig subclass groups, we analyzed relationships of IgGsum and IgG, performed backward stepwise regression on IgG and D using appropriate variables, computed correlations of IgG1 and IgG2, and determined IgG subclass proportions of IgGsum. We discuss the present results in the context of factors that influence IgG and IgG subclass levels in adults with IgGSD.

## Results

### 39 adults with subnormal IgG1 only

General characteristics. There were 35 women (89.7%). IgG < 7.00 g/L was observed in 15 patients (38.5%). Clinical attributes of patients with IgG < 7.00 g/L and ≥ 7.00 g/L were similar, except that the prevalence of AC was significantly lower in patients with IgG < 7.00 g/L (Table 1). Mean IgG was significantly lower in patients with IgG < 7.00 g/L, by definition. Median IgG1 and median IgG2 were also significantly lower in patients with IgG < 7.00 g/L (Table 1).

**Table 1:** Characteristics of 39 adults with IgGSD and subnormal IgG1 only^a^

Characteristics	IgG < 7.00 g/L, % (n = 15)	IgG ≥ 7.00 g/L, % (n = 24)	Value of *p*
Women, % (n)	100.0 (15)	83.3 (20)	0.1458
Mean age at diagnosis, y ± 1 SD	53 ± 15	45 ± 14	0.1196
Autoimmune condition(s), % (n)^b^	26.7 (4)	62.5 (15)	0.0484
Atopy, % (n)^c^	33.3 (5)	20.8 (5)	0.4633
Autoimmune condition(s) and atopy, % (n)	3.3 (2)	4.2 (1)	0.5470
Mean IgG, g/L ± 1 SD	6.10 ± 0.57	8.19 ± 0.85	< 0.0001
Median IgG1, g/L (range)	3.21 (2.82, 4.12)	3.89 (3.27, 4.18)	< 0.0001
Median IgG2, g/L (range)	2.28 (1.25, 3.92)	3.04 (1.66, 5.96)	0.0147
Median IgG3, g/L (range)	0.55 (0.41, 1.14)	0.66 (0.43, 1.38)	0.1057
Median IgG4, g/L (range)	0.12 (0.01, 0.86)	0.16 (0.01, 0.54)	0.9424
Mean IgA, g/L ± 1 SD	1.74 ± 0.82	1.91 ± 0.73	0.5374
Median IgM, g/L (range)	1.18 (0.50, 4.08)	0.96 (0.40, 2.79)	0.3054

^a^IgGSD, IgG subclass deficiency; SD, standard deviation. Subnormal IgG1 is defined as a value < 2 SD below the mean. No patient had subnormal serum levels of IgG2, IgG3, IgG4, IgA, or IgM. One of 35 women (2.9%) had elevated IgG3. Six of 35 women (17.1%) had elevated IgM. Neither elevated IgG3 nor elevated IgM was observed in the four men represented herein

^b^Autoimmune condition(s) were diagnosed in 19 patients: ankylosing spondylitis (2); autoimmune thyroiditis (9); Behçet disease (1); Graves disease (2); inflammatory arthritis (1); myasthenia gravis (1); pernicious anemia (1); psoriasis (2); rheumatoid arthritis (1); Sjögren syndrome (3); systemic lupus erythematosus (2); and undifferentiated connective tissue disorder (2). Seven of the 19 patients (36.8%) had two or more autoimmune conditions

^c^Atopy was diagnosed in 10 patients (7 allergic asthma, 4 allergic rhinitis, 1 allergic dermatitis). Two of the 10 patients (20.0%) had two atopy conditions

#### IgG and IgGsum

The difference between mean IgGsum and mean IgG was not significant (7.34 ± 1.31 g/L vs. 7.39 ± 1.27 g/L, respectively; *p* = 0.8816). The correlation of IgGsum and IgG was significant (Pearson correlation coefficient = 0.8063; adjusted r^2^ = 0.6406; *p* < 0.0001). Mean IgGsum was lower in patients with IgG < 7.00 g/L than IgG ≥ 7.00 g/L (6.40 ± 0.84 g/L vs. 7.93 ± 1.22 g/L, respectively; *p* < 0.0001). Mean D was also lower in patients with IgG < 7.00 g/L than IgG ≥ 7.00 g/L (− 11.0 ± 5.4 vs. 4.9 ± 8.7, respectively; *p* < 0.0001). Thus, the mean difference in IgG and IgGsum in adults with subnormal IgG1 only subgrouped by IgG1 levels (< 7.00 g/L vs. ≥ 7.00 g/L) was ~ 15.9%.

#### Regressions

Backward stepwise regression on IgG using age at diagnosis, sex, and IgG subclasses as independent variables revealed two positive associations: IgG1 (*p* < 0.0001) and IgG2 (*p* < 0.0001) (ANOVA *p* < 0.0001). This model accounted for 71.1% of the deviance of IgG. Backward stepwise regression on D using age at diagnosis and sex as independent variables revealed no significant associations.

### 53 adults with combined subnormal IgG1/IgG3

General characteristics. There were 47 women (88.7%). IgG < 7.00 g/L was observed in 32 patients (60.4%). Clinical attributes of patients with IgG < 7.00 g/L and ≥ 7.00 g/L were similar (Table 2). Mean IgG was significantly lower in patients with IgG < 7.00 g/L, by definition. Mean IgG1 and mean IgG2 were significantly lower in patients with IgG < 7.00 g/L (Table 2).

**Table 2:** Characteristics of 53 adults with IgGSD and combined subnormal IgG1/IgG3^a^

Characteristics	IgG < 7.00 g/L, % (n = 32)	IgG ≥ 7.00 g/L, % (n = 21)	Value of *p*
Women, % (n)	93.8 (30)	50.9 (17)	0.1995
Mean age at diagnosis, y ± 1 SD	52 ± 14	50 ± 10	0.5653
Autoimmune condition(s), % (n)^b^	28.1 (9)	38.1 (8)	0.5511
Autoimmune condition(s) and atopy, % (n)	0	0	1.0000
Atopy, % (n)^c^	31.3 (10)	38.1 (8)	0.4633
Mean IgG, g/L ± 1 SD	6.07 ± 0.68	8.73 ± 1.20	< 0.0001
Mean IgG1, g/L ± 1 SD	3.11 ± 0.55	3.70 ± 0.41	< 0.0001
Mean IgG2, g/L ± 1 SD	2.41 ± 0.67	4.02 ± 1.15	< 0.0001
Mean IgG3, g/L ± 1 SD	0.29 ± 0.09	0.31 ± 0.07	0.4188
Median IgG4, g/L (range)	0.21 (0.02, 0.49)	0.21 (0.01, 0.88)	0.5010
Median IgA, g/L (range)	1.77 (0.86, 13.09)	1.94 (1.26, 4.47)	0.2713
Median IgM, g/L (range)	0.98 (0.41, 4.07)	1.19 (0.45, 4.15)	0.6364

^a^IgGSD, IgG subclass deficiency; SD, standard deviation. Subnormal IgG1/IgG3 is defined as values < 2 SD below the respective means. No patient had subnormal serum levels of IgG2, IgG4, IgA, or IgM. Three of 47 women (6.4%) had elevated IgA. Six of 47 women (12.8%) had elevated IgM. Neither elevated IgG3 or elevated IgM was observed in the six men represented herein

^b^Autoimmune condition(s) were diagnosed in 17 patients: ankylosing spondylitis (1); autoimmune thyroiditis (9); Graves disease (1); multiple sclerosis (1); pernicious anemia (1); polymyalgia rheumatica (1); rheumatoid arthritis (2); Sjögren syndrome (1); and undifferentiated connective tissue disorder (1). One of the 18 patients (5.6%) had two autoimmune conditions

^c^Atopy was diagnosed in 18 patients (15 allergic asthma, 3 allergic rhinitis, 2 allergic dermatitis). Two of the 18 patients (11.1%) had two atopy conditions

#### IgG and IgGsum

Mean IgGsum and mean IgG did not differ significantly (6.94 ± 1.51 g/L vs. 7.12 ± 1.60 g/L, respectively; *p* = 0.5392). The correlation of IgG and IgGsum was significant (Pearson correlation coefficient = 0.9064; adjusted r^2^ = 0.8181; *p* < 0.0001). Mean IgGsum was lower in patients with IgG < 7.00 g/L than IgG ≥ 7.00 g/L (6.04 ± 0.73 g/L vs. 8.61 ± 1.35 g/L, respectively (*p* < 0.0001). Median D in patients with IgG < 7.00 g/L and those with IgG ≥ 7.00 g/L did not differ significantly (− 2.2 (− 32.3, 21.8) vs. − 2.7 (− 30.9, 9. 9), respectively; *p* = 0.1561).

#### Regressions

Backward stepwise regression on IgG using age at diagnosis, sex, and IgG subclasses as independent variables revealed two positive associations: IgG1 (*p* < 0.0001) and IgG2 (*p* < 0.0001) (ANOVA *p* < 0.0001). This model accounted for 82.5% of the deviance of IgG. Backward stepwise regression on D using age at diagnosis and sex as independent variables revealed no significant association.

### 115 patients with IgGSD and subnormal IgG3 only

General characteristics. Mean age of these patients was 47 ± 13 y. There were 105 women (90.3%). Clinical and laboratory attributes are displayed in Table 3.

**Table 3:** Characteristics of 115 adults with IgGSD and subnormal IgG3 only^a^

Women, % (n)	87.8 (105)
Mean age at diagnosis, y ± 1 SD	47 ± 13
Autoimmune condition(s), % (n)^b^	31.3 (36)
Atopy, % (n)^c^	27.8 (32)
Autoimmune condition(s) and atopy, % (n)	6.1 (7)
Mean IgG, g/L ± 1 SD	10.00 ± 1.87
Median IgG1, g/L (range)	5.77 (4.23, 10.70)
Mean IgG2, g/L ± 1 SD	3.23 ± 1.20
Mean IgG3, g/L ± 1 SD	0.28 ± 0.08
Median IgG4, g/L (range)	0.23 (0.01, 2.37)
Median IgA, g/L (range)	1.85 (0.71, 5.55)
Median IgM, g/L (range)	1.19 (0.43, 5.16)

^a^IgGSD, IgG subclass deficiency; SD, standard deviation. Subnormal IgG3 is defined as a value < 2 SD below the mean. No patient had subnormal serum levels of IgG1, IgG2, IgG4, IgA, or IgM. Six of 101 women (5.9%) had elevated IgA. Sixteen of 101 women (15.8%) had elevated IgM. Neither elevated IgA nor elevated IgM was observed in the ten men represented herein

^b^Autoimmune condition(s) were diagnosed in 36 patients: anti-nuclear antibody > 1:80 without other explanation (5); anti-phospholipid antibody syndrome (1); autoimmune thyroiditis (2); Behçet syndrome (1); Crohn’s disease (2); Graves disease (1); mixed connective tissue disorder (1); psoriasis (3); psoriatic arthritis (2); rheumatoid arthritis (8); sarcoidosis (1); Sjögren syndrome (6); systemic lupus erythematosus (3); ulcerative colitis (1); and vitiligo (1)

^c^Atopy was diagnosed in 32 patients (22 allergic asthma; 10 allergic rhinitis; 3 allergic eczema). Three of the 32 patients (9.4%) had two atopy conditions

#### IgG and IgGsum

The difference between mean IgGsum and mean IgG was not significant (9.71 ± 2.01 g/dL vs. 10.00 ± 1.87 g/L, respectively; *p* = 0.2680). The correlation of IgGsum and IgG was significant (Pearson correlation coefficient = 0.8859; adjusted r^2^ = 0.7828; *p* < 0.0001).

#### Regressions

Backward stepwise regression on IgG using age at diagnosis, sex, and IgG subclasses as independent variables revealed two positive associations: IgG1 (*p* < 0.0001) and IgG2 (*p* < 0.0001) (ANOVA *p* < 0.0001). This model accounted for 80.7% of the deviance of IgG. Backward stepwise regression on D using age at diagnosis and sex as independent variables revealed no significant association.

### IgG subclasses as percentages of IgGsum

IgG1 percentages of IgGsum were significantly lower and IgG2 percentages of IgGsum were significantly higher in patients with subnormal IgG1 subclass levels than in patients with subnormal IgG3 only (Table 4). Other comparisons of these data are displayed in Table 4, along with presentation of IgG subclass percentages of IgGsum in ostensibly healthy adults in four studies from the literature (Table 4). We also compared ratios of levels of IgG1, IgG2, and IgG3 subclasses to that of IgG4 for each subject group. This revealed that the ratio of IgG2: IgG4 is higher for the three present subject groups than has been reported in previous studies of ostensibly healthy adults (Table 4).

**Table 4:** IgG subclasses as percentages of IgGsum^a^

Subjects	IgG1, %^c^	IgG2, %^d^	IgG3, %^e^	IgG4, %^f^	Reference
Adults with IgGSD and subnormal IgG1 alone (n = 39)	50.2 ± 8.3	37.9 ± 8.7	8.1 (5.2, 17.3)	2.0 (0.2, 11.7)	Present study
*Ratios of subclasses compared to IgG4*	*25.1*	*19.0*	*4.1*	*1.0*	
Adults with IgGSD and combined subnormal IgG1/IgG3 (n = 53)	49.2 ± 8.4	42.8 ± 9.1	4.3 (1.7, 7.1)	2.7 (0.2, 9.6)	Present study
*Ratios of subclasses compared to IgG4*	*18.2*	*15.9*	*1.6*	*1.0*	
Adults with IgGSD and subnormal IgG3 alone (n = 115)^b^	61.3 ± 8.1	32.7 ± 8.1	2.9 (0.7, 5.4)	2.4 (0.1, 12.4)	Present study
*Ratios of subclasses compared to IgG4*	*25.5*	*13.6*	*1.2*	*1.0*	
Normal adults (n = 108)^b^	62.8	30.5	2.4	4.4	[28]
*Ratios of subclasses compared to IgG4*	*14.3*	*6.9*	*0.5*	*1.0*	
Laboratory personnel (n = 107)^b^	58.4	31.3	5.4	4.9	[29]
*Ratios of subclasses compared to IgG4*	*11.9*	*6.4*	*1.1*	*1.0*	
Healthy adults (n = 172)^b^	60.3	31.0	6.2	2.5	[2]
*Ratios of subclasses compared to IgG4*	*24.1*	*12.4*	*2.5*	*1.0*	
Blood donors (n = 100)^b^	61.7	30.3	5.7	2.3	[30]
*Ratios of subclasses compared to IgG4*	*26.8*	*13.2*	*2.5*	*1.0*	

Each row of italics corresponds to the non-italics font entries above

^a^IgGsum = sum of subclass levels (IgG1 + IgG2 + IgG3 + IgG4); IgGSD, Ig subclass deficiency. Data from the present study are expressed as mean ± 1 SD or median (range) of individual IgG subclass measurements as proportions of IgGsum (± 1 standard deviation)

^b^Data cited from the literature are expressed as the aggregate mean IgG subclass level as a proportion of the sum of aggregate mean total IgG subclass (IgG1 + IgG2 + IgG3 + IgG4) values

^c^Mean percent IgG1 in both subnormal IgG1 subclass groups is lower than mean percent IgG1 in subnormal IgG3 group (*p* < 0.0001, both comparisons). The difference in mean percent IgG1 between the two subnormal IgG1 subclass groups is not significant (*p* = 0.5856)

^d^Mean percent IgG2 in subnormal IgG1 only group is lower than mean percent IgG2 in combined subnormal IgG1/IgG3 group (*p* = 0.0106). Mean percent IgG2 in subnormal IgG3 group is lower than mean percent IgG2 in either subnormal IgG1 subclass group (*p* < 0.0001, both comparisons)

^e^Median percent IgG3 is higher in subnormal IgG1 group than median percent IgG3 in combined subnormal IgG1/IgG3 or subnormal IgG3 groups (*p* < 0.0001, both comparisons). Median percent IgG3 in combined subnormal IgG1/IgG3 group is higher than that in subnormal IgG3 group (*p* < 0.0001)

^f^Median percent IgG4 is lower in subnormal IgG1 group than in combined subnormal IgG1/IgG3 group (*p* = 0.0377). There is no significant difference in median percent IgG in subnormal IgG1 only and subnormal IgG3 only groups (*p* = 0.4634). Median percent IgG4 in combined subnormal IgG1/IgG3 group is higher than that in subnormal IgG3 group (*p* = 0.0453)

### Correlations of IgG1 and IgG2

**Fig. 1:**
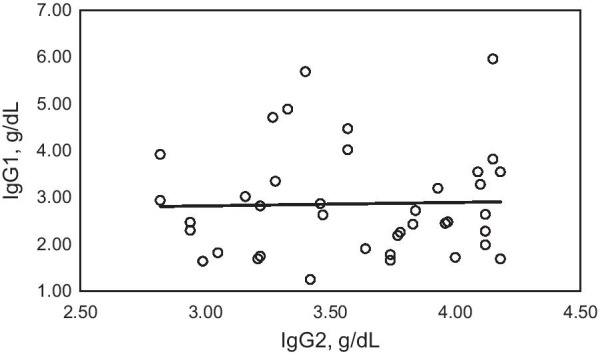
Correlation of IgG1 and IgG2 in 39 adult patients with IgGSD and subnormal IgG1 only. Pearson correlation coefficient 0.0277 (adjusted r^2^ =  ~ 0; *p* = 0.8816)

**Fig. 2:**
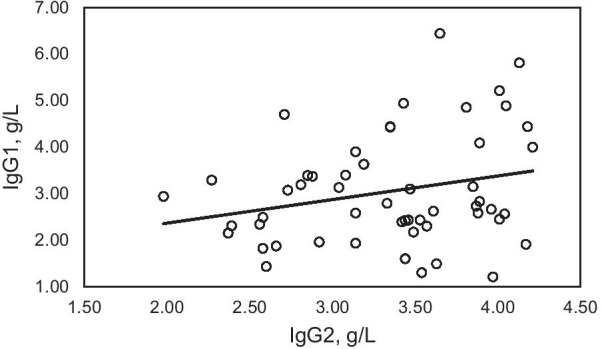
Correlation of IgG1 and IgG2 in patients with 53 adult patients with IgGSD and combined subnormal IgG1/IgG3. Pearson correlation coefficient 0.2456 (adjusted r^2^ = 0.0419; *p* = 0.0762)

**Fig. 3:**
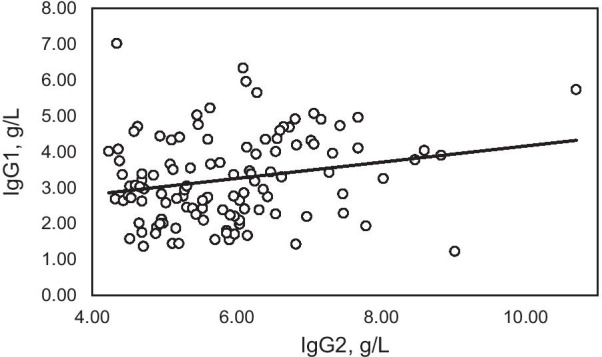
Correlation of IgG1 and IgG2 in 115 adults patients with IgGSD and subnormal IgG3 only. Pearson correlation coefficient 0.2185 (adjusted r^2^ = 0.0393; *p* = 0.0190)

In patients with subnormal IgG1 only, the Pearson correlation coefficient of IgG1 vs. IgG2 was 0.0277 (adjusted r^2^ =  ~ 0; *p* = 0.8816) (Fig. 1). In patients with combined subnormal IgG1/IgG3, the Pearson correlation coefficient of IgG1 vs. IgG2 was 0.2456 (adjusted r^2^ = 0.0419; *p* = 0.0762) (Fig. 2). In patients with subnormal IgG3 only, the Pearson correlation coefficient of IgG1 vs. IgG2 was 0.2185 (adjusted r^2^ = 0.0393; *p* = 0.0190) (Fig. 3).

### Elevated IgA in IgGSD patients

Elevated IgA levels (> 4.00 g/L, > 2 SD above the mean) were observed only in women (Tables 1, 2 and 3). Altogether, nine women (4.8%) and none of 20 men had elevated IgA (*p* = 0.6047).

### Elevated IgM in IgGSD patients

Elevated IgM levels (> 2.30 g/L, > 2 SD above the mean) were observed only in women (Tables 1, 2 and 3). Altogether, 28 of 187 women (15.0%) and none of 20 men had elevated IgM (*p* < 0.0001).

## Discussion

This study of adults with IgGSD demonstrates that both IgG1 and IgG2 are major determinants of IgG in patients with subnormal IgG1, combined subnormal IgG1/IgG3, or subnormal IgG3, after adjustment for other variables. In patients with subnormal IgG1 or combined subnormal IgG1/IgG3, we also demonstrate that median IgG2 levels are significantly lower in those with IgG < 7.00 g/L than those with IgG ≥ 7.00 g/L. In the present study, IgG < 7.00 g/L occurred in 39% of patients with subnormal IgG1 only and 60% of patients with combined subnormal IgG1/IgG3. These latter observations are consistent with two respective reports that IgG was subnormal (< 7.00 g/L) in 38% and 46% of adults with IgGSD who had subnormal IgG1 only without other subnormal IgG subclasses, subnormal IgA, or subnormal IgM [5, 6], contradicting previous suggestions that patients with low IgG1 will most likely be identified without IgG subclass testing [31, 32].

In the present patients with subnormal IgG1 subclass patterns, mean IgG1 was significantly lower in those with IgG < 7.00 g/L than IgG ≥ 7.00 g/L. Regressions on IgG revealed significant positive associations of IgG1, both in patients with subnormal IgG1 only and patients with combined subnormal IgG1/IgG3, after adjustment for other variables. In 12 adult patients with IgGSD and subnormal IgG2 and subnormal IgG, nine also had subnormal IgG1 [16]. These observations demonstrate that IgG1 is a major component of IgG in adults with IgGSD as it is in subjects unselected for subnormal IgG subclass levels [2, 4, 28–30].

IgG2 levels in the present patients were within reference limits, by definition. Regardless, mean or median IgG2 levels in patients with subnormal IgG1 subclass levels were significantly lower in patients with IgG < 7.00 g/L than in patients with IgG ≥ 7.00 g/L. Regressions on IgG revealed significant positive associations of IgG2 in both subnormal IgG1 subclass groups, after adjustment for other variables. In 12 adults with IgGSD selected for subnormal IgG2 levels who also had subnormal IgG, three had normal IgG1 [16]. These observations demonstrate that IgG2 is also a major component of IgG in adults with IgGSD as it is in normal subjects [1, 2, 4, 28–30]. In contrast, none of 115 adults with IgGSD and subnormal IgG3 only had subnormal IgG [20].

In patients with subnormal IgG1, the prevalence of AC was significantly greater in those with IgG ≥ 7.00 g/L than IgG < 7.00 g/L. This suggests that preferential synthesis of IgG1, IgG2, or both may occur as a consequence of one or more AC. In another study, respective mean serum levels of IgG1 were significantly higher and mean serum levels of IgG2 were significantly lower in patients with primary Sjögren syndrome (pSS), systemic lupus erythematosus (SLE), and systemic sclerosis than in healthy controls [33]. Two other studies found that serum IgG1, IgG2, and IgG3 levels were significantly higher in patients with pSS [34] and SLE [35] than in normal control subjects.

IgG1 percentages of IgGsum were significantly lower and IgG2 percentages of IgGsum were significantly higher in patients with subnormal IgG1 subclass levels than in patients with subnormal IgG3 only. Correlations of IgG1 and IgG2 values in patients with subnormal IgG1 subclass levels were not significant. Although the correlation of IgG1 and IgG2 was significant in patients with subnormal IgG3 only, the strength of the Pearson correlation coefficient was low. Taken together, these observations suggest that a) IgG1 and IgG2 subclass proportions of IgGsum and absence of significant correlation of IgG1 and IgG2 in the present patients with subnormal IgG1 subclass levels are consequences of the subnormal IgG1 selection criteria we used; and/or that b) that synthesis of IgG1 and IgG2 in adults with IgGSD and subnormal IgG1 subclass levels is not regulated in tandem.

IgG1, IgG2 and IgG3 molecules have polymorphic antigens known as Gm (gamma marker) allotypes on the constant regions of their respective γ1, γ2, and γ3 heavy chains encoded in *IGHG* loci (chromosome 14q32.33**)** [36, 37]. Some Gm allotypes are associated with different serum Ig levels. For example, the “normal” IgG2 range for persons with allotype G2m(23)- is 35% lower than that of persons with G2m(23) + [38]. There was a three-fold difference across mean serum IgG3 levels of normal adults grouped by G3m allotypes [29]. Few persons have deletions or other structural changes in *IGHG* loci that decrease the level of one or more IgG subclasses [39–43]. Most patients with IgGSD have dysfunctional regulation of IgG subclass production [44]. Intravascular distributions, fractional catabolic rates, and average biologic half-lives of IgG1 and IgG2 are similar [3].

All patients in this study presented with frequent or recurrent upper or lower respiratory tract infection and some of them were discovered to have subnormal IgG1 subclass levels. Adults in two other studies also had frequent or recurrent upper or lower respiratory tract infection and subnormal IgG1 only [5, 6]. In a California cohort of 78 adults with IgGSD, 27 (35%) had subnormal IgG1 (< 3.42 g/L), alone or in combination with subnormal IgG3 or IgG4 [21]. In a study of 3005 persons ≥ one year of age who had frequent or severe episodes of infection (and their relatives) and other patients without infection discovered incidentally to have hypogammaglobulinemia, 119 (4%) had subnormal IgG1 only [45]. Of these 119 patients, 83% had infections, especially sinusitis [45]. These and related observations [46] suggest that the proportion of adults with subnormal IgG1 who have or eventually develop frequent or severe respiratory tract infection is high.

Mean IgG2 was significantly lower in patients with IgG < 7.00 g/L than IgG ≥ 7.00 g/L in this study, although the IgG2 level of each patient was within the reference limit. Subnormal IgG2 is associated with frequent or severe respiratory infection in some persons [13–16], whereas other persons with subnormal or absent IgG2 are healthy [30, 40, 41, 47, 48]. Thus, it is unknown whether quantitative differences in IgG2 levels in the present patients contributed to their frequent or severe respiratory tract infection.

IgG3 exerts multiple effector functions against many viral and bacterial pathogens [1, 49]. Subnormal IgG3 only is the most common IgGSD pattern in adults [19–21, 25]. Nonetheless, IgG3 levels < 2 SD between population means are common in ostensibly healthy adults [2, 28–30] and in some patient groups unselected for frequent or severe respiratory tract infection or subnormal Ig [50–52].

The mean difference in IgG and IgGsum in the present adults with subnormal IgG1 only subgrouped by IgG1 levels (< 7.00 g/L vs. IgG ≥ 7.00 g/L) was ~ 16%. In another study, IgG and IgGsum differed by > 15% in 11% of 571 consecutive clinical samples [53]. The difference between IgG and IgGsum correlated with the proportion but not level of IgG1 [53]. After dilution of samples with differences > 15%, repeat testing did not reduce the differences significantly [53]. In the present study, we did not observe significant mean differences in D in adults with combined subnormal IgG1/IgG3 subgrouped by IgG1 levels (< 7.00 g/L vs. IgG ≥ 7.00 g/L).

Infection susceptibility was increased in persons with common AC [54–58] and atopy [59–62] who were unselected for IgGSD. The odds of respiratory tract infection were significantly increased in male Finnish military recruits with mannose-binding lectin levels below the median, after adjustment for asthma status [63]. In contrast, the prevalence of mannose-binding lectin ≤ 50 µg/L in white adults with IgGSD did not differ significantly from that in general European white populations [64]. In persons with subnormal IgE, the prevalence of frequent or severe respiratory tract infection [65, 66], other subnormal Ig isotypes [65, 67], and autoimmune conditions [66, 67] was significantly greater than that of control subjects. Hypogammaglobulinemia E in adults with IgGSD was negatively associated with bronchitis, allergic asthma, IgG1, and levels of blood CD4 + lymphocytes, after adjustment for other variables [68].

In this study, there was a predominance of women in all IgGSD groups, consistent with other reports of IgGSD in adults [6, 16, 19–21, 24, 45, 69]. The predominance of females among persons with IgGSD becomes evident at puberty [70]. AC are common in adults with IgGSD [6, 16, 19–21, 45] and women predominate among adults with AC [71, 72]. The prevalence of chronic rhinosinusitis in women, the most common respiratory tract infection in adults with IgGSD [21, 64], is twice that of men [73]. Taken together, it is plausible that factors related to the X-chromosome, X-chromosome inactivation, or hormonal differences between women and men could explain the predominance of women in cohorts of adults with IgGSD, although this is unproven.

Elevated IgA occurred in 5% of 187 women and no men in this study, although the difference in prevalence was not significant. Elevated IgA occurs in some patients with AC [74, 75] and others with cirrhosis due to chronic hepatitis B [76]. In healthy women and men grouped by age, there was no significant difference in mean IgA levels [77].

Elevated IgM occurred in 15% of 187 women and none of 20 men in this study. Elevated IgM levels are common in AC [78]. In 404 adults in the U.S. ages 20–89 y without conditions known to affect Ig levels, women had significantly higher IgM levels than men, regardless of age [77]. In a population-based survey of 460 adults in Spain, there was a significant negative association of male sex with IgM levels, after adjustment for other variables [79]. Serum IgM levels in adults are directly related to the number of X-chromosomes [80].

The present regression analyses demonstrate that IgG1 and IgG2 levels account for 71–82% of the variance of IgG levels in 207 adults with IgGSD. Thus, we infer that other factors not included in our analyses also influence IgG levels. We cannot exclude the possibility that AC, atopy, IgA, or IgM levels also influence IgG, although that possibility is not suggested by the results of the present univariate analyses. IgG subclass data from individual age- and sex-matched control subjects unselected for frequent or severe upper or lower respiratory tract infection or IgGSD were not available for analysis. Results in other IgGSD cohorts could vary due to referral differences and between-laboratory variation in methods of analysis, control data, and consequent IgG and IgG subclass reference limits. Ascertaining Gm allotypes, studying specific antibody activity, quantifying IgGSD responses to PPSV, and measuring IgE and mannose-binding lectin levels were beyond the scope of this work.

## Conclusions

We conclude that both IgG1 and IgG2 are major determinants of IgG in patients with subnormal IgG1, combined subnormal IgG1/IgG3, or subnormal IgG3 and that in patients with subnormal IgG1 or combined subnormal IgG1/IgG3, median IgG2 levels are significantly lower in those with IgG < 7.00 g/L than those with IgG ≥ 7.00 g/L.

## Methods

### IgGSD definition

IgGSD was defined as frequent or severe respiratory tract infection, one or more subnormal IgG subclass level(s) (< 2 SD below respective means) unexplained by other causes, and decreased IgG response to PPSV [20–25]. Frequent respiratory tract infection was defined as four or more episodes per year that required antibiotic therapy. Severe respiratory tract infection was defined as any respiratory tract infection that required in-hospital treatment [81]. All patients were evaluated and diagnosed to have IgGSD during the interval November 1991–December 2019 using the same criteria [16, 20].

### Patient selection

We reviewed the medical records of consecutive, unrelated, self-identified non-Hispanic white adults (ages ≥ 18 y) referred to a single outpatient hematology clinic (Southern Hematology & Oncology, P.C., Birmingham, AL, USA) because they had frequent or severe upper or lower respiratory tract infection inadequately managed with antibiotic and ancillary therapy, were subsequently diagnosed to have IgGSD, and had no first-degree relatives with IgGSD or other Ig deficiency previously known to this clinic as described in detail previously [16, 20, 25, 82]. Referring physicians diagnosed AC and atopy (allergic asthma, allergic rhinitis, allergic dermatitis/eczema). There are no Hispanic adults with IgGSD in our clinic population.

### Patient exclusions

We excluded patients who reported therapy with daily oral corticosteroids [16]. We also excluded patients: who reported taking hydroxychloroquine [83–86]; captopril, carbamazepine, chloroquine, diphenylhydantoin, fenclofenac, gold, hydantoin, levamisole, penicillamine, sulfasalazine, valproic acid, or zonisamide [85]; oxcarbazapine [87]; leflunomide; methotrexate; or rituximab; with subnormal IgG2 [16] or subnormal IgA, or subnormal IgM; with monoclonal gammopathy; and with subnormal IgG subclass, subnormal IgA, or subnormal IgM levels due to other defined causes [16]. We also excluded patients with subnormal IgG4 because: (1) we desired to avoid introducing an additional IgG subclass variable; (2) substantial minorities of healthy men and women do not have detectable IgG4 [2, 88]; and (3) IgG4 levels measured by our laboratory that are > 2 SD below the lower reference limit are reported as zero.

### Laboratory methods

IgG at diagnosis in each patient was measured using turbidimetry (Laboratory Corporation of America, Burlington, NC, USA). IgG subclasses were measured using four separate quantifications on corresponding specimens using rate nephelometry (Laboratory Corporation of America, Burlington, NC, USA) and reported separately from IgG. IgG and IgG subclasses were measured before IgG replacement therapy was initiated. For all Ig measurements, we defined mean ± 2 SD as reference limits [6, 89, 90]: IgG 7.00–16.00 g/L; IgG1 4.22–12.92 g/L; IgG2 1.17–7.47 g/L; IgG3 0.41–1.29 g/L; IgG4 0.01–2.91 g/L (0–2.9 g/L); IgA 0.70–4.00 g/L; and IgM 0.40–2.30 g/L. Ig reference ranges for diagnosis of IgGSD did not change during the interval 1991–2021. Subnormal Ig levels were defined as those below the corresponding lower reference limits. Subnormal Ig levels were documented twice in all adults at times they did not have acute infection. We used the second IgG subclass values for the present analyses.

### Statistical analysis

Data for analyses consisted of observations on 207 adults: 39 with subnormal IgG1 only; 53 with combined subnormal IgG1/IgG3; and 115 with subnormal IgG3 only (see Additional file 1: Supplemental File "Observations in 207 Adults with IgGSD"). We evaluated continuous data for normality using d'Agostino's and Shapiro–Wilk tests. Descriptive data are displayed as enumerations, percentages, means (± 1 SD), or medians (range). Age and serum Ig data are expressed to the nearest integer. To determine factors that affect the percentage difference between IgGsum and IgG, we computed the D-parameter [27], defined as:

$$ D = \frac{IgGsum - IgG}{{IgGsum}} \cdot 100\% , $$

where IgGsum is the sum of IgG subclasses (IgG1 + IgG2 + IgG3 + IgG4) and IgG is total serum IgG.

Mean values of data from normal distributions were compared using Student's t test (two-tailed). Differences in median values of data from non-normal distributions were compared using the Mann–Whitney U test. We compared differences in proportions of dichotomous variables using Fisher's exact test (two-tailed). We compared some continuous data using Pearson’s correlation coefficient. We ranked the strength of statistically significant positive Pearson correlation coefficients as low (0.1–0.3), medium (> 0.3–0.5), and large (> 0.5–1.0).

The number of observations in each of the present IgGSD patient groups was relatively small and thus we minimized the numbers of independent variables we used in regression analyses [91]. For regressions on IgG, we included IgG subclass values and also age and sex because some IgG subclass levels in healthy adults are significantly influenced by these latter variables [88, 92]. For regressions on D, we used only age and sex as independent variables.

We defined values of *p* < 0.05 to be significant. Analyses were performed with Excel 2000® (Microsoft Corp., Redmond, WA, USA) and GB-Stat® (v. 10.0, 2003, Dynamic Microsystems, Inc., Silver Spring, MD, USA).

## Supplementary Information

**Additional file 1**. Observations in 207 Adults with IgGSD.

## Data Availability

All data generated or analysed during this study are included in this published article [and its Additional file 1].

## Abbreviations

AC: Autoimmune condition(s)
D: D-parameter
IgGsum: Sum of IgG subclasses
Ig: Immunoglobulin
IgG: Immunoglobulin G
IgGSD: IgG subclass deficiency
PPSV: Pneumococcal polysaccharide vaccination
SD: Standard deviation
SLE: Systemic lupus erythematosus
pSS: Primary Sjögren syndrome

## References

[1] Vidarsson G, Dekkers G, Rispens T (2014). IgG subclasses and allotypes: from structure to effector functions. Front Immunol.

[2] French MA, Harrison G (1984). Serum IgG subclass concentrations in healthy adults: a study using monoclonal antisera. Clin Exp Immunol.

[3] Morell A, Terry WD, Waldmann TA (1970). Metabolic properties of IgG subclasses in man. J Clin Invest.

[4] Jefferis R, Kumararatne DS (1990). Selective IgG subclass deficiency: quantification and clinical relevance. Clin Exp Immunol.

[5] Van Kessel DA, Horikx PE, Van Houte AJ, De Graaff CS, Van Velzen-Blad H, Rijkers GT (1999). Clinical and immunological evaluation of patients with mild IgG1 deficiency. Clin Exp Immunol.

[6] Barton JC, Bertoli LF, Barton JC, Acton RT. Selective subnormal IgG1 in 54 adult index patients with frequent or severe bacterial respiratory tract infections. J Immunol Res. 2016;1405950. 10.1155/2016/1405950 PMC4830719 27123464

[7] Hashira S, Okitsu-Negishi S, Yoshino K (2000). Placental transfer of IgG subclasses in a Japanese population. Pediatr Int.

[8] Stapleton NM, Brinkhaus M, Armour KL, Bentlage AEH, de Taeye SW, Temming AR (2019). Reduced FcRn-mediated transcytosis of IgG2 due to a missing glycine in its lower hinge. Sci Rep.

[9] Siber GR, Schur PH, Aisenberg AC, Weitzman SA, Schiffman G (1980). Correlation between serum IgG-2 concentrations and the antibody response to bacterial polysaccharide antigens. N Engl J Med.

[10] Barrett DJ, Ayoub EM (1986). IgG2 subclass restriction of antibody to pneumococcal polysaccharides. Clin Exp Immunol.

[11] Kojima K, Ishizaka A, Oshika E, Taguchi Y, Tomizawa K, Nakanishi M (1990). Quantitation of IgG subclass antibodies to pneumococcal capsular polysaccharides by ELISA, using Pneumovax-specific antibodies as a reference. Tohoku J Exp Med.

[12] Schauer U, Stemberg F, Rieger CH, Buttner W, Borte M, Schubert S (2003). Levels of antibodies specific to tetanus toxoid, *Haemophilus influenzae* type b, and pneumococcal capsular polysaccharide in healthy children and adults. Clin Diagn Lab Immunol.

[13] Oxelius VA (1974). Chronic infections in a family with hereditary deficiency of IgG2 and IgG4. Clin Exp Immunol.

[14] Braconier JH, Nilsson B, Oxelius VA, Karup-Pedersen F (1984). Recurrent pneumococcal infections in a patient with lack of specific IgG and IgM pneumococcal antibodies and deficiency of serum IgA, IgG2 and IgG4. Scand J Infect Dis.

[15] Stanley PJ, Corbo G, Cole PJ (1984). Serum IgG subclasses in chronic and recurrent respiratory infections. Clin Exp Immunol.

[16] Barton JC, Barton JC, Bertoli LF, Acton RT (2020). Characterization of adult patients with IgG subclass deficiency and subnormal IgG2. PLoS ONE.

[17] Morell A, Skvaril F, Steinberg AG, Van LE, Terry WD (1972). Correlations between the concentrations of the four sub-classes of IgG and Gm Allotypes in normal human sera. J Immunol.

[18] Garty BZ, Ludomirsky A, Danon YL, Peter JB, Douglas SD (1994). Placental transfer of immunoglobulin G subclasses. Clin Diagn Lab Immunol.

[19] Abrahamian F, Agrawal S, Gupta S (2010). Immunological and clinical profile of adult patients with selective immunoglobulin subclass deficiency: response to intravenous immunoglobulin therapy. Clin Exp Immunol.

[20] Barton JC, Bertoli LF, Barton JC, Acton RT (2016). Selective subnormal IgG3 in 121 adult index patients with frequent or severe bacterial respiratory tract infections. Cell Immunol.

[21] Khokar A, Gupta S (2019). Clinical and immunological features of 78 adult patients with primary selective IgG subclass deficiencies. Arch Immunol Ther Exp (Warsz).

[22] Soderström T, Soderström R, Avanzini A, Brandtzaeg P, Karlsson G, Hanson LA (1987). Immunoglobulin G subclass deficiencies. Int Arch Allergy Appl Immunol.

[23] Aghamohammadi A, Lougaris V, Plebani A, Miyawaki T, Durandy A, Hammarström L: Predominantly antibody deficiencies**.** Edited by Rezaei N, Aghamohammadi A, Notarangelo LD. Berlin: Springer-Verlag; 2008:97–130.

[24] Kim JH, Park HJ, Choi GS, Kim JE, Ye YM, Nahm DH (2010). Immunoglobulin G subclass deficiency is the major phenotype of primary immunodeficiency in a Korean adult cohort. J Korean Med Sci.

[25] Barton JC, Bertoli LF, Barton JC (2014). Comparisons of CVID and IgGSD: referring physicians, autoimmune conditions, pneumovax reactivity, immunoglobulin levels, blood lymphocyte subsets, and HLA-A and -B typing in 432 adult index patients. J Immunol Res.

[26] Schwartz RA, Lin RY, Vafale J, Berger M. Immunoglobulin G deficiency. Edited by Kaliner MA. Medscape. Updated 6-12-2020. Accessed 7-7-2020. https://emedicine.medscape.com/article/136897-overview

[27] Pasternak G, Lewandowicz-Uszynska A, Pentos K (2018). Analysis of differences between total IgG and sum of the IgG subclasses in children with suspected immunodeficiency—indication of determinants. BMC Immunol.

[28] Morell A, Skvaril F: A modified radioimmunoassay for quantitative determination of IgG subclasses in man**.** In *Protides of Biological Fluids (vol. 19)*. Edited by Peeters H. London: 1971:533–540.

[29] van der Giessen M, Rossouw E, van Veen TA, Van LE, Zegers BJ, Sander PC (1975). Quantification of IgG subclasses in sera of normal adults and healthy children between 4 and 12 years of age. Clin Exp Immunol.

[30] Madassery JV, Kwon OH, Lee SY, Nahm MH (1988). IgG2 subclass deficiency: IgG subclass assays and IgG2 concentrations among 8015 blood donors. Clin Chem.

[31] Marsh RA, Orange JS (2019). Antibody deficiency testing for primary immunodeficiency: a practical review for the clinician. Ann Allergy Asthma Immunol.

[32] Maguire GA, Kumararatne DS, Joyce HJ (2002). Are there any clinical indications for measuring IgG subclasses?. Ann Clin Biochem.

[33] Zhang H, Li P, Wu D, Xu D, Hou Y, Wang Q (2015). Serum IgG subclasses in autoimmune diseases. Medicine (Baltimore).

[34] Liu Y, Li J (2011). Preferentially immunoglobulin (IgG) subclasses production in primary Sjogren's syndrome patients. Clin Chem Lab Med.

[35] Lin GG, Li JM (2009). IgG subclass serum levels in systemic lupus erythematosus patients. Clin Rheumatol.

[36] Lefranc MP, Lefranc G (2012). Human Gm, Km, and Am allotypes and their molecular characterization: a remarkable demonstration of polymorphism. Methods Mol Biol.

[37] Review of the notation for the allotypic and related markers of human immunoglogulins. J Immunol. 1976;117:1056–1058. 956649

[38] Nahm MH, Macke K, Kwon OH, Madassery JV, Sherman LA, Scott MG. Immunologic and clinical status of blood donors with subnormal levels of IgG2. J Allergy Clin Immunol. 1990;85:769–77. 10.1016/0091-6749(90)90197-c 2324414

[39] Cox DW, Markovic VD, Teshima IE (1982). Genes for immunoglobulin heavy chains and for alpha 1-antitrypsin are localized to specific regions of chromosome 14q. Nature.

[40] Lefranc MP, Lefranc G, Rabbitts TH (1982). Inherited deletion of immunoglobulin heavy chain constant region genes in normal human individuals. Nature.

[41] Lefranc G, Chaabani H, van Loghem E, Lefranc MP, de Gerda L, Helal AN (1983). Simultaneous absence of the human IgG1, IgG2, IgG4 and IgA1 subclasses: immunological and immunogenetical considerations. Eur J Immunol.

[42] Migone N, Oliviero S, de Gerda L, Delacroix DL, Boschis D, Altruda F (1984). Multiple gene deletions within the human immunoglobulin heavy-chain cluster. Proc Natl Acad Sci USA.

[43] Keyeux G, Lefranc G, Lefranc MP (1989). A multigene deletion in the human *IGH* constant region locus involves highly homologous hot spots of recombination. Genomics.

[44] Pan Q, Hammarström L (2000). Molecular basis of IgG subclass deficiency. Immunol Rev.

[45] Lacombe C, Aucouturier P, Preud'homme JL (1997). Selective IgG1 deficiency. Clin Immunol Immunopathol.

[46] Aucouturier P, Lacombe C, Preud'homme JL (1994). Serum IgG subclass level determination: methodological difficulties and practical aspects. Ann Biol Clin.

[47] Hammarström L, Smith CI (1983). IgG2 deficiency in a healthy blood donor. Concomitant lack of IgG2, IgA and IgE immunoglobulins and specific anti-carbohydrate antibodies. Clin Exp Immunol.

[48] Kuijpers TW, Weening RS, Out TA (1992). IgG subclass deficiencies and recurrent pyogenic infections, unresponsiveness against bacterial polysaccharide antigens. Allergol Immunopathol (Madr ).

[49] Damelang T, Rogerson SJ, Kent SJ, Chung AW (2019). Role of IgG3 in infectious diseases. Trends Immunol.

[50] Barton JC, Bertoli LF, Acton RT (2003). Common variable immunodeficiency and IgG subclass deficiency in central Alabama hemochromatosis probands homozygous for *HFE* C282Y. Blood Cells Mol Dis.

[51] Bertoli LF, Pappas DG, Barton JC, Barton JC (2014). Serum immunoglobulins in 28 adults with autoimmune sensorineural hearing loss: increased prevalence of subnormal immunoglobulin G1 and immunoglobulin G3. BMC Immunol.

[52] Barton JC, Barton JC, Cruz E, Teles MJ, Guimaraes JT, Porto G (2020). Chromosome 6p SNP microhaplotypes and IgG3 levels in hemochromatosis probands with HFE p.C282Y homozygosity. Blood Cells Mol Dis.

[53] McLean-Tooke A, O'Sullivan M, Easter T, Loh R (2013). Differences between total IgG and sum of the IgG subclasses in clinical samples. Pathology.

[54] Doran MF, Crowson CS, Pond GR, O'Fallon WM, Gabriel SE (2002). Frequency of infection in patients with rheumatoid arthritis compared with controls: a population-based study. Arthritis Rheum.

[55] Grijalva CG, Chen L, Delzell E, Baddley JW, Beukelman T, Winthrop KL (2011). Initiation of tumor necrosis factor-alpha antagonists and the risk of hospitalization for infection in patients with autoimmune diseases. JAMA.

[56] Danza A, Ruiz-Irastorza G (2013). Infection risk in systemic lupus erythematosus patients: susceptibility factors and preventive strategies. Lupus.

[57] Listing J, Gerhold K, Zink A (2013). The risk of infections associated with rheumatoid arthritis, with its comorbidity and treatment. Rheumatology (Oxford).

[58] Quartuccio L, Zabotti A, Del ZS, Zanier L, De VS, Valent F (2019). Risk of serious infection among patients receiving biologics for chronic inflammatory diseases: usefulness of administrative data. J Adv Res.

[59] Juhn YJ, Kita H, Yawn BP, Boyce TG, Yoo KH, McGree ME (2008). Increased risk of serious pneumococcal disease in patients with asthma. J Allergy Clin Immunol.

[60] Jung JA, Kita H, Yawn BP, Boyce TG, Yoo KH, McGree ME (2010). Increased risk of serious pneumococcal disease in patients with atopic conditions other than asthma. J Allergy Clin Immunol.

[61] Rantala A, Jaakkola JJ, Jaakkola MS (2013). Respiratory infections in adults with atopic disease and IgE antibodies to common aeroallergens. PLoS ONE.

[62] Juhn YJ (2014). Risks for infection in patients with asthma (or other atopic conditions): is asthma more than a chronic airway disease?. J Allergy Clin Immunol.

[63] Rantala A, Lajunen T, Juvonen R, Bloigu A, Silvennoinen-Kassinen S, Peitso A (2008). Mannose-binding lectin concentrations, *MBL2* polymorphisms, and susceptibility to respiratory tract infections in young men. J Infect Dis.

[64] Barton JC, Barton JC, Bertoli LF (2019). Clinical and laboratory associations of mannose-binding lectin in 219 adults with IgG subclass deficiency. BMC Immunol.

[65] Smith JK, Krishnaswamy GH, Dykes R, Reynolds S, Berk SL (1997). Clinical manifestations of IgE hypogammaglobulinemia. Ann Allergy Asthma Immunol.

[66] Magen E, Schlesinger M, Ben-Zion I, Vardy D (2015). *Helicobacter pylori* infection in patients with selective immunoglobulin E deficiency. World J Gastroenterol.

[67] McVicker S, Karim MY (2014). IgE deficiency may indicate underlying hypogammaglobulinaemia?. J Clin Pathol.

[68] Barton JC, Barton JC, Bertoli LF (2017). Hypogammaglobulinemia E in 216 adults with IgG subclass deficiency and respiratory tract infections. Ann Allergy Asthma Immunol.

[69] Bjorkander J, Bengtsson U, Oxelius VA, Hanson LA (1986). Symptoms in patients with lowered levels of IgG subclasses, with or without IgA deficiency, and effects of immunoglobulin prophylaxis. Monogr Allergy.

[70] Björkander J, Bengtsson U, Oxelius VA, Hanson LA (1986). Symptoms in patients with lowered levels of IgG subclasses, with or without IgA deficiency, and effects of immunoglobulin prophylaxis. Monogr Allergy.

[71] Angum F, Khan T, Kaler J, Siddiqui L, Hussain A (2020). The prevalence of autoimmune disorders in women: a narrative review. Cureus.

[72] Dinse GE, Parks CG, Weinberg CR, Co CA, Wilkerson J, Zeldin DC (2020). Increasing prevalence of antinuclear antibodies in the United States. Arthritis Rheumatol.

[73] Ference EH, Tan BK, Hulse KE, Chandra RK, Smith SB, Kern RC (2015). Commentary on gender differences in prevalence, treatment, and quality of life of patients with chronic rhinosinusitis. Allergy Rhinol (Providence ).

[74] Badcock LJ, Clarke S, Jones PW, Dawes PT, Mattey DL (2003). Abnormal IgA levels in patients with rheumatoid arthritis. Ann Rheum Dis.

[75] Zhao EJ, Carruthers MN, Li CH, Mattman A, Chen LYC (2020). Conditions associated with polyclonal hypergammaglobulinemia in the IgG4-related disease era: a retrospective study from a hematology tertiary care center. Haematologica.

[76] Lin S, Sun Q, Mao W, Chen Y (2016). Serum immunoglobulin A (IgA) level Is a potential biomarker indicating cirrhosis during chronic hepatitis B infection. Gastroenterol Res Pract.

[77] Crisp HC, Quinn JM (2009). Quantitative immunoglobulins in adulthood. Allergy Asthma Proc.

[78] Duarte-Rey C, Bogdanos DP, Leung PS, Anaya JM, Gershwin ME (2012). IgM predominance in autoimmune disease: genetics and gender. Autoimmun Rev.

[79] Gonzalez-Quintela A, Alende R, Gude F, Campos J, Rey J, Meijide LM (2008). Serum levels of immunoglobulins (IgG, IgA, IgM) in a general adult population and their relationship with alcohol consumption, smoking and common metabolic abnormalities. Clin Exp Immunol.

[80] Rhodes K, Markham RL, Maxwell PM, Monk-Jones ME (1969). Immunoglobulins and the X-chromosome. Br Med J.

[81] Barton J, Barton C, Bertoli L (2019). Duration of frequent or severe respiratory tract infection in adults before diagnosis of IgG subclass deficiency. PLoS ONE.

[82] Bousfiha A, Jeddane L, Picard C, Ailal F, Bobby GH, Al-Herz W (2018). The 2017 IUIS phenotypic classification for primary immunodeficiencies. J Clin Immunol.

[83] Fox RI, Chan E, Benton L, Fong S, Friedlaender M, Howell FV (1988). Treatment of primary Sjögren's syndrome with hydroxychloroquine. Am J Med.

[84] Lege-Oguntoye L, Onyemelukwe GC, Maiha BB, Udezue EO, Eckerbom S (1990). The effect of short-term malaria chemoprophylaxis on the immune response of semi-immune adult volunteers. East Afr Med J.

[85] Hammarström L, Vorechovsky I, Webster D (2000). Selective IgA deficiency (SIgAD) and common variable immunodeficiency (CVID). Clin Exp Immunol.

[86] Bodewes ILA, Gottenberg JE, van Helden-Meeuwsen CG, Mariette X, Versnel MA (2020). Hydroxychloroquine treatment downregulates systemic interferon activation in primary Sjögren's syndrome in the JOQUER randomized trial. Rheumatology (Oxford).

[87] Knight AK, Cunningham-Rundles C (2005). Oxcarbazepine-induced immunoglobulin deficiency. Clin Diagn Lab Immunol.

[88] Aucouturier P, Mounir S, Preud'homme JL (1985). Distribution of IgG subclass levels in normal adult sera as determined by a competitive enzyme immunoassay using monoclonal antibodies. Diagn Immunol.

[89] Barton JC, Bertoli LF, Acton RT (2003). HLA-A and -B alleles and haplotypes in 240 index patients with common variable immunodeficiency and selective IgG subclass deficiency in central Alabama. BMC Med Genet.

[90] Schauer U, Stemberg F, Rieger CH, Borte M, Schubert S, Riedel F (2003). IgG subclass concentrations in certified reference material 470 and reference values for children and adults determined with the binding site reagents. Clin Chem.

[91] Smith G (2018). Step away from stepwise. J Big Data.

[92] De Greef GE, Van Tol MJ, Van Den Berg JW, Van Staalduinen GJ, Janssen CJ, Radl J (1992). Serum immunoglobulin class and IgG subclass levels and the occurrence of homogeneous immunoglobulins during the course of ageing in humans. Mech Ageing Dev.

[93] World Medical Association (2013). Declaration of Helsinki: ethical principles for medical research involving human subjects. JAMA.

